# OLDER DRIVER REPORTS OF EXPERIENCE WITH SENSORS IN VEHICLES TO RECORD DRIVING BEHAVIOR

**DOI:** 10.1093/geroni/igae098.2904

**Published:** 2024-12-31

**Authors:** Kelley Jackson, Ruth Tappen, David Newman, Jinwoo Jang, Borko Furht, KwangSoo Yang, Mónica Rosselli, Michelle Villar

**Affiliations:** Florida Atlantic University, Boca Raton, Florida, United States; Florida Atlantic University, Boca Raton, Florida, United States; Florida Atlantic University, Boca Raton, Florida, United States; Florida Atlantic University, Boca Raton, Florida, United States; Florida Atlantic University, Boca Raton, Florida, United States; Florida Atlantic University, Boca Raton, Florida, United States; Florida Atlantic University, Boca Raton, Florida, United States; Florida Atlantic University, Boca Raton, Florida, United States

## Abstract

In a 5-year study of the degree to which changes in driving behavior herald a change in cognition, participants were asked to evaluate the telematic and video sensors (both driver facing and forward facing) installed in their vehicles in terms of any distraction or other concerns the sensors may have created. Both an 8-item survey and open-ended questions were asked of study participants every three months for the duration of their enrollment (three years). The questions concerned acceptability of sensors in one’s vehicle including the aesthetics, source of distraction and potential for invasion of privacy. Mean overall rating of acceptability of the sensors was 3.5 out of a possible high score of 5. This rating was 3.5 at baseline, increased to 3.7 at the end of 6 months [t(110)=1.09, p=.004]. and was maintained over time. Of some interest is that there were no statistically significant differences in acceptability ratings by gender, race, ethnicity, age or education. A general inductive approach was employed to code the qualitative responses. The majority were concerned with the wires connecting sensors to power sources, both their visibility and tendency to loosen over time, creating both aesthetic and operational concerns as well as a tripping hazard for some. These were addressed early in the study to the satisfaction of most participants. The size of both the sensors and wires was mentioned a number of times and several participants suggested using smaller, wireless sensors if possible. Overall, most participants quickly acclimated to having sensors in their vehicles.

